# Genomics, Molecular Taxonomy, and Functional Genetics: Insights from a Diverse Collection of Studies

**DOI:** 10.3390/genes17050501

**Published:** 2026-04-24

**Authors:** Pedro Lorite, Antonio Figueras

**Affiliations:** 1 Departamento de Biología Experimental, Universidad Jaén, 23071 Jaén, Spain; 2 Instituto de Investigaciones Marinas (IIM-CSIC), 36208 Vigo, Spain

This Special Issue of *Genes* brings together a set of contributions that, despite their apparent thematic diversity, share a common methodological thread: the application of genomic and molecular tools to fundamental questions in animal biology, ranging from production traits in livestock to phylogenetic identity in wild species. The seven articles published here collectively illustrate both the breadth and integrative power of modern genomics when applied across taxa and biological levels of organization.

## 1. Genomics of Productive Traits in Livestock

Two studies address the genetic architecture of commercially relevant traits in cattle. Borges Dos Reis et al. [1] revisit a methodological question with practical consequences: whether assuming equal variance across SNPs in genome-wide association studies (GWASs) based on single-step genomic BLUP (ssGBLUP) is adequate for complex traits related to meat quality in beef cattle. Their findings demonstrate that alternative weighting approaches can improve the identification of genomic windows associated with these traits, reinforcing the case for model flexibility in livestock GWAS. This work adds to a growing body of evidence suggesting that the biological architecture of complex traits is not well captured by uniform-variance assumptions [2,3].

The second livestock contribution, by Sasazaki et al. [4], adopts a finer-resolution approach, moving from genomic windows to candidate causal polymorphisms. Focusing on the fat area ratio of the rib eye in Japanese Black cattle, a trait tightly correlated with Beef Marbling Standard scores, the authors identify candidate variants in *GGT1* and *SLC5A1* on BTA17, thereby providing a pathway from the association signal to a functional hypothesis. The identification of causal polymorphisms rather than associated genomics regions remains one of the central challenges in animal genomics, and this study represents a methodologically careful step in that direction.

## 2. Molecular Taxonomy and Cryptic Diversity

A recurring theme in this Special Issue is the capacity of molecular data to reveal taxonomic complexity that is invisible to morphological analysis. Vella et al. [5] provide a striking example from the Maltese islands, demonstrating through molecular taxonomy that the gecko fauna of this archipelago includes a second *Tarentola* species previously unrecognized. The Maltese islands occupy a biogeographically significant position in the Sicilian Channel, and this finding has direct implications for conservation policy and for understanding colonization routes across the central Mediterranean. The study exemplifies how molecular tools continue to reshape our understanding of vertebrate diversity even in apparently well-studied regions.

Similarly, Rico-Porras et al. [6] apply cytogenomic approaches to flea beetles of the genus *Podagrica* (Coleoptera, Chrysomelidae), characterizing the karyotype and the satellitome in a group notable for its unusually high chromosome number within the Chrysomelidae family. The integration of classical cytogenetics with genomic data (cytogenomics) represents a maturing approach that bridges two historically separate disciplines, and the detailed satellitome analysis presented here contributes to a still fragmentary picture of repetitive element diversity in beetles.

## 3. Historical and Ancient DNA

The contribution by Pasicka et al. [7] extends genomic analysis into the archaeological record, presenting mitochondrial DNA genomes from early medieval horse remains excavated across Silesia (present-day Poland). Ancient DNA studies of domestic animals provide a window into the genetic diversity and movement patterns of historical populations that are otherwise inaccessible. The mtDNA data reported here complement the growing European DNA datasets and contribute to the reconstruction of the breeding and trading networks of medieval horse populations in Central Europe.

## 4. Functional Genetics and Gene Regulation

Two studies address gene regulation and expression at the functional level. Krasnov et al. [8] characterize the transcriptome of sterile testes in *dnd*-depleted Atlantic salmon (*Salmo salar*), identifying genes involved in both gonadal and brain development in the context of reproductive sterility induced by primordial germ cell depletion. The approach has both basic and applied relevance: understanding the transcriptomic consequences of sterility is central to aquaculture programs that seek to prevent genetic introgression from farmed fish into wild populations.

Cosenza et al. [9] address a different regulatory mechanism, reviewing the evidence for genetically modulated alternative splicing in two casein genes, *CSN1S1* and *CSN1S2*. The identification of allele-specific splicing events driven by SNPs and indels in regulatory sequences has both evolutionary and practical implications, particularly for milk protein composition and quality in dairy species. This review consolidates evidence that splicing regulatory sequences are more polymorphic and functionally consequential than previously recognized.

## 5. Conclusions

Taken together, the contributions to this Special Issue reflect the current state of animal genomics: methodologically diverse, taxonomically broad, and increasingly capable of connecting molecular variation to biological function. From production traits in domesticated cattle to cryptic species in Mediterranean geckos, from medieval horse populations to sterile transgenic salmon, the common thread is the use of genomic and molecular tools to address questions that were inaccessible a decade ago.

We thank all authors for their contributions, the reviewers for their rigorous evaluation of each manuscript, and the editorial team of *Genes* for their support throughout the preparation of this Special Issue.
